# Sexual and Gender Minority Status and Suicide Mortality: An Explainable Artificial Intelligence Analysis

**DOI:** 10.3389/ijph.2024.1606855

**Published:** 2024-05-06

**Authors:** Ying Yin, T. Elizabeth Workman, John R. Blosnich, Cynthia A. Brandt, Melissa Skanderson, Yijun Shao, Joseph L. Goulet, Qing Zeng-Treitler

**Affiliations:** ^1^Washington DC VA Medical Center, United States Department of Veterans Affairs, Washington, DC, United States; ^2^ Biomedical Informatics Center, The George Washington University, Washington, DC, United States; ^3^ Center for Health Equity Research and Promotion, VA Pittsburgh Healthcare System, Veterans Health Administration, United States Department of Veterans Affairs, Pittsburgh, PA, United States; ^4^Suzanne Dworak-Peck School of Social Work, University of Southern California, Los Angeles, CA, United States; ^5^ VA Connecticut Healthcare System, Veterans Health Administration, United States Department of Veterans Affairs, West Haven, CT, United States; ^6^Pain, Research, Informatics, Multi-Morbidities, and Education Center, VA Connecticut Healthcare System, West Haven, CT, United States

**Keywords:** sexual and gender minority, suicide mortality, risk factors, deep learning, explainable artificial intelligence

## Abstract

**Objectives:** Suicide risk is elevated in lesbian, gay, bisexual, and transgender (LGBT) individuals. Limited data on LGBT status in healthcare systems hinder our understanding of this risk. This study used natural language processing to extract LGBT status and a deep neural network (DNN) to examine suicidal death risk factors among US Veterans.

**Methods:** Data on 8.8 million veterans with visits between 2010 and 2017 was used. A case-control study was performed, and suicide death risk was analyzed by a DNN. Feature impacts and interactions on the outcome were evaluated.

**Results:** The crude suicide mortality rate was higher in LGBT patients. However, after adjusting for over 200 risk and protective factors, known LGBT status was associated with reduced risk compared to LGBT-Unknown status. Among LGBT patients, black, female, married, and older Veterans have a higher risk, while Veterans of various religions have a lower risk.

**Conclusion:** Our results suggest that disclosed LGBT status is not directly associated with an increase suicide death risk, however, other factors (e.g., depression and anxiety caused by stigma) are associated with suicide death risks.

## Introduction

According to the United States Center for Disease Control, “suicide is a leading cause of death in the United States, with 47,646 deaths in 2021.” [1] Suicide risk, in terms of suicidal ideation and suicide attempt, is particularly elevated in people who identify as sexual and gender minority, including lesbian, gay, bisexual, and transgender (LGBT) identities [2–5].

One of the many challenges in research on suicidal thoughts and behaviors is that outcomes are relatively rare and multi-determined [6]. The causes of suicidal behaviors may consist of a combination of factors, which vary between individuals and groups over time. As Mościcki noted, risk may be distal or proximal in time to the event [7]. Distal factors, such as past trauma, may increase risk in conjunction with changes in proximal risk factor(s), such as re-traumatization. More proximal factors can include sudden life disruptions (e.g., job loss, relationship failures) [8] and dynamic factors in the symptomology of serious mental illnesses such as major depressive disorder, bipolar disorder, and schizophrenia [9]. Importantly, although sexual and gender minority populations have greater burdens of suicidal thoughts and behaviors [10], in this study, we maintain that LGBT status is not a direct risk factor, rather it is associated with other factors that increases suicide risk. Principal among these related risks is minority stress [11], which operates at several levels including interpersonally (e.g., family rejection); social adversities such as homelessness and victimization; fears of engaging in healthcare due to system- and provider-level issues such as discrimination and bias [12]. Minority stress may contribute to or interact with other proximal risk factors for suicide such as comorbid mental health and medical conditions (e.g., depression, chronic pain) [6, 13]. A better understanding requires novel analyses of variable interactions and identification of new factors [14].

A critical barrier to better understand suicide risk and its correlates among LGBT individuals is the overall lack of structured data on sexual orientation and gender identity (SO/GI) in healthcare administrative and clinical data systems, such as electronic health records (EHR). Despite recent progress in SO/GI data collection in some health systems [15], there remain large gaps. For example, in the US, the Centers for Disease Control and Prevention (CDC) National Syndromic Surveillance Program is a crucial source of data to monitor suicide attempts necessitating medical treatment [16]. However, because most EHR systems do not have structured SO/GI data fields (or if they do the fields are largely missing data) [17], LGBT people are not visible in data reported by emergency departments. Consequently, as EHR systems continue to develop and implement SO/GI fields, researchers have turned to machine learning innovations to develop health services research for LGBT patients [18–20].

In addition to limited administrative data on suicidal ideation and suicide attempt, information about suicide mortality of LGBT patients is even more scant. Because SO/GI data are not collected at the time of death [21], no vital statistics systems include data to learn about LGBT mortality. The CDC’s National Violent Death Reporting System (NVDRS) is one example, however, it only focuses on violent death and SO/GI data are missing for approximately 80% of decedents in the data repository [22]. Recent efforts using EHR data have attempted to estimate suicide mortality for LGBT individuals, but these efforts have used either solely diagnosis-based efforts to identify patients [4] or used idiosyncratic machine learning techniques [5]. As a nascent, heterogeneous field, inquiry into LGBT suicide mortality needs more study to help determine nuances in methodology and corroboration of findings.

EHR data also offer opportunities to examine many potential risk factors for suicide, including demographic and clinical variables [23]. Temporal information in longitudinal records provides additional information by allowing researchers to differentiate an event in the past from one more proximal to the event of interest. Moreover, risk factors for suicide are likely to interact with each other and do not necessarily have a linear relationship with the outcome. A type of deep neural network (DNN) model called transformer is one potential form of analysis to identify and explore sexual and gender minority statuses and suicide risk. DNN has the capability of modeling non-linear relationships and complex interactions between covariates [24]. Transformer, in particular, has demonstrated superior capability in modeling sequence data such as temporal history [25].

A trained DNN model is sometimes viewed as a “black box” as it can be difficult for humans to interpret, partly due to the large number of hyperparameters. Fortunately, in recent years, researchers including the present team have developed explainable artificial intelligence (AI) methods to enable the estimation of feature contribution and feature interaction [26, 27]. Using these methods, we can identify risk factors and assess interactions in a similar fashion as in traditional statistical analyses.

For the present analyses, we utilized EHR data from the US Department of Veteran Affairs (VA) Corporate Data Warehouse (CDW). Over 20 years ago, the VA, which operates the largest healthcare system in the U.S., adopted a comprehensive “paperless” EHR [28] that is integrated system-wide. Data from 2000 onward is stored in the CDW, making it readily accessible for clinical research. Thus the VA EHR provides a unique laboratory in which to conduct observational research from a host of structured and unstructured data [29]. We identified a cohort of patients had or had not died by suicide and their disclosed LGBT status were identified using an NLP algorithm [30]. The objective of the study is to assess the impact of disclosed LGBT status on suicide death using DNN model and explainable AI.

## Methods

### Data Source

The data source was the VA CDW.

### Cohort

The cohort was defined as Veterans who had at least one outpatient encounter between Fiscal Year (FY) 2010 and FY 2017 (*n* = 8,827,824). The first outpatient visit date is defined as the enrollment date. National death index (NDI) data were used to obtain primary and underlying causes and date of death for the veterans (observed until 31st December 2018). Suicide death (*n* = 21,942) was defined based on ICD-10 codes (U03, X60-84 and Y87.0) according to the underlying cause of death reported by the NDI [31].

### Study Design

We took a case-control approach for this study because our primary interest is in the risk factors rather than prediction. The cases were patients in the cohort who died by suicide before calendar year 2018. Veterans without suicide death were matched 1:1 without replacement to the cases by fiscal year of enrollment (i.e., the fiscal year they entered the cohort) and endpoint. The endpoint of the case is the date of death and endpoint of the control is the first outpatient visit date of the year matching the case’s endpoint. The endpoint date is set as index date for case and control. We thus identified 21,942 case-control pairs.

### Nature Language Processing

We used a previously developed NLP application to extract the LGBT status of veterans from their clinic notes [30]. The application uses a hybrid pipeline of rule-based and machine learning methods. First, it extracts clinical notes with key phrases related to LGBT status, such as “gay,” “transgender,” “bisexual,” and “lesbian.” Next, it uses regular expressions to scan for negative and positive patterns of these terms. Documents that do not match these patterns are further classified by a trained machine learning random forest model. The application achieved 88.2% sensitivity, 91.5% specificity, and 85.9% positive predictive value. We used clinic notes from 01 October 1999 to 26 January 2021 for our cohort to identify the LGBT-Yes and LGBT-No status for our cohort. The LGBT status of patients with no relevant notes is unknown.

### Feature Groups

Based on prior research [32, 33] and the goal of our research, we compiled a compressive list of 203 predictors of suicide death reflecting patient demographics, socioeconomic status, physical and mental health history:

1) Socio-demographics: age, EHR-based sex, race/ethnicity, marital status, religion.
2) LGBT status: based on NLP algorithm.
3) History of prior suicidal behaviors: suicide attempts and suicide ideation based on ICD-9-CM and ICD-10-CM codes reported in inpatient and outpatient visits. The visit date of the diagnosis documented is used as the feature date (same for all the diagnoses below).
4) Diagnosis of mental health disorders: depression, anxiety, PTSD, bipolar disorder, substance abuse, and sleep disorder, etc., based on ICD-9-CM and ICD-10-CM codes reported in inpatient and outpatient visits.
5) History of physical illness: pain, fractures, diabetes, sex dysfunction, etc. based on ICD-9-CM and ICD-10-CM codes reported in inpatient and outpatient visits.
6) History of VA medication fills: prescription fill records within the VA systems are grouped by drug categories. The drug categories are based on VA drug classes and their pharmacology effects. Only drug categories with a prevalence of 0.5% in the cohort are included as features. Prescription fill dates are used as the feature date.
7) History of documented firearm access: firearm access history is based on keyword search and NLP prediction of the clinic notes. The document reference date is used as the feature date.
8) History of homelessness: This was determined using homeless stop code (a type of visit code in VA data), homeless ICD codes, Inpatient bed section codes (indicating the type of hospitalization), and the VA’s universal screening for housing instability [34].
9) History of hospitalization. The inpatient admission date is used as the feature date.

### Variables and Values

The socio-demographic variables and LGBT status are represented as static data. Age is calculated at the index date. The multivalued categorical variables are converted to n-1 binary variables.

For the temporal features, we divided the history into 21 time-periods. From 10 years to 7 days prior to the index date, we had 20 time-slides each with the length of 180 days; 10 years prior and beyond was grouped as a preceding, one time-period. The length of the time period was chosen empirically. If the feature date falls within the time-period, the presence of the feature is represented with a feature id and time-period id. If the patient does not have any recorded medical history for a time-period, no data were associated with that time-period id. The 7-day buffer is used to exclude data on the suicide event itself.

The outcome variable, which is the suicide death, is a binary variable taking the values of 1/0 representing the presence/absence of the adverse outcome.

### DNN Model

**FIGURE 1:**
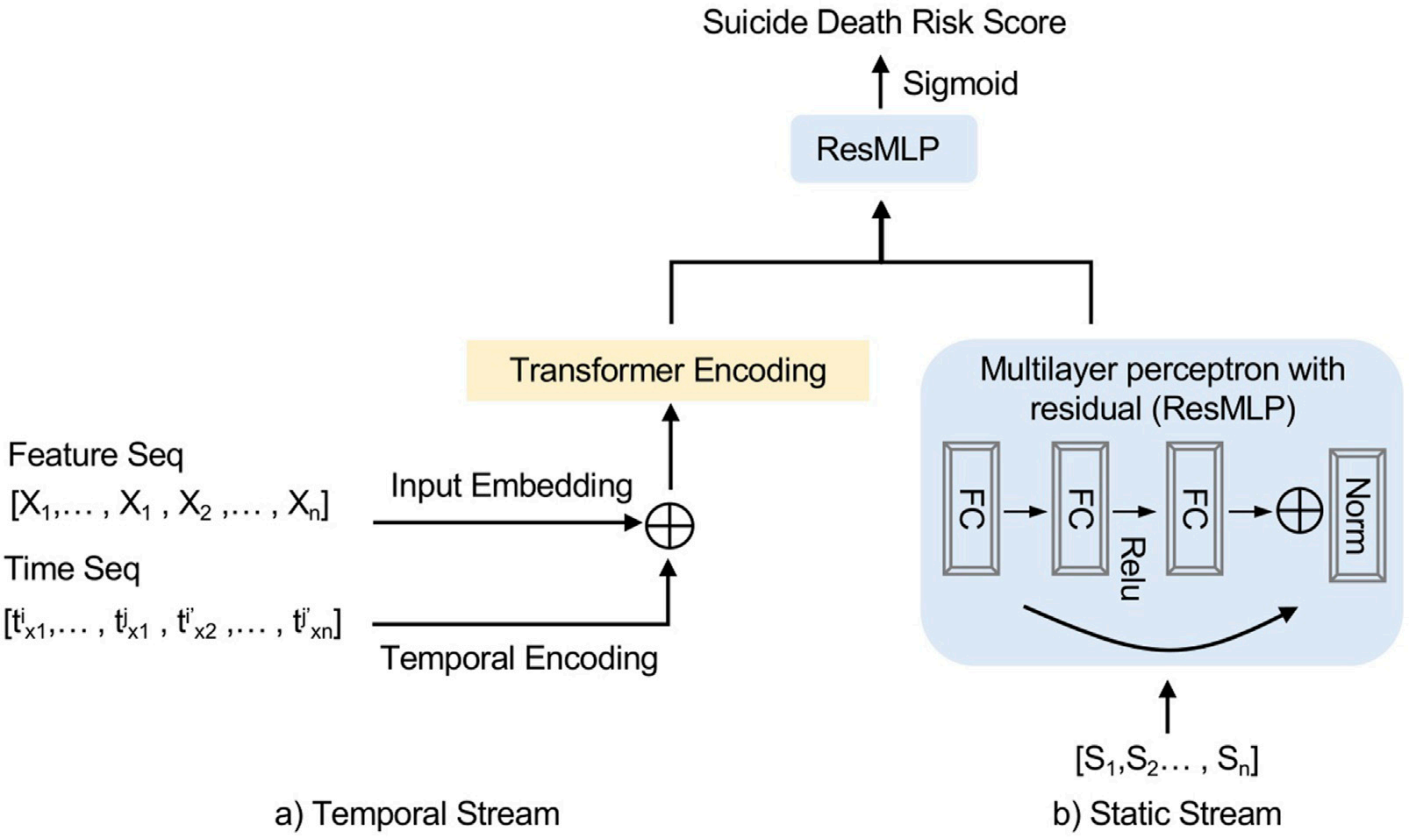
Deep neural network model architecture. X_n_ represents the temporal feature and t_xn_ represents the time period when X_n_ presents in data. S_n_ represent the static features and FC represents a fully connected feedforward layer (United States, 2023).

The architecture of the DNN model is shown in Figure 1. There are two streams of input layers: the temporal stream and the static stream as prepared in the section above.

The temporal stream combined the feature inputs and time inputs after embedding and encoded with transformer encoder. The transformer encoder was assembled from the original transformer architecture with two blocks of encoder layers. The static stream encoded the static features with a multilayer perceptron block with residual. Then the two streams were combined by element-wise addition. The sigmoid function σ served as the activation function so that the output value was between 0 and 1. The output values were called risk scores, as higher values corresponded to higher risks of the adverse outcome. For model training, we computed the binary-cross-entropy function as the loss function and the area under ROC curve (AUC) was used as the main metric for measuring the model performance.

### Impact and Impact Scores

To evaluate the impact of input features in the DDN model, we calculated the impact/impact scores based on a method developed by our team. The detailed definitions of the scores have been previously published [27, 35]. Briefly, the scores measure the impact of changing a feature value to a reference value on the output risk score. The absolute value of the score represents the magnitude of impact and the “plus-minus” sign indicates the direction of the impact: a positive impact indicates a higher risk, and a negative score indicates a lower risk.

For each variable, an individual-level impact/impact score is defined as:

$$\text{impact}=\text{logit }\left(p_{\text{cur}}\right)‐\text{logit}\left(p_{\text{ref}}\right)$$

$$\text{impact score}=\text{impact }/\;\left(\text{current value}‐\text{reference value}\right)$$

Where p_cur_ is the output from the DNN model and p_ref_ is new value of p after mutation. For the binary variables and temporal variables, we calculate the impact as the reference values as either 0 or absence. For patient’s age, the impact score is calculated to measure the impact of a 10-year change in age from the reference age (Average Age of the cohort = 61.6).

### Interaction and Interaction Score

To measure the interaction of the variables with LGBT status, we calculate the interaction score [27, 35] by comparing the impact of varying the values of both variables and the impacts of varying the variables separately. The residual impact is considered as the interaction between the two variables. A positive interaction score indicates a higher combined risk than the sum of the risks of the two features involved, while a negative score indicates a lower combined risk.

## Result

Among, the 8.8 million veterans, we found 200,834 (2.3%) veterans with disclosed LGBT status and 437,689 (5.0%) veterans with disclosed non-LGBT status from clinic notes. The rest had unknown LGBT status. Before matching, patients with disclosed LGBT status had lower all-cause mortality rates (13% LGBT, 15% non-LGBT, 23% LGBT-unknown) (Table 1). Veterans with disclosed LGBT status were more likely to die from suicide (2.2% of overall all-cause mortality) than those with non-LGBT status (1.8%) and LGBT-unknown (1.1%) veterans. These findings are consistent with previous research [36].

**TABLE 1:** All-cause mortality rate and suicide mortality rate among Veterans healthcare users between fiscal year 2010 and 2017 based on the LGBT status (United States, 2023).

	Total patients	All-cause mortality^a^	Suicide mortality	All-cause mortality rate (%)	Suicide mortality rate (%)	Suicide mortality over all-cause mortality (%)
All	8,817,264	1,941,798	21,942	22	0.25	1.1
LGBT-Unknown	8,178,741	1,848,295	20,166	23	0.25	1.1
LGBT-Yes	200,834	25,944	575	13	0.29	2.2
LGBT-No	437,689	67,559	1,201	15	0.27	1.8

^a^ Patient’s death status and cause of death are from national death index data observed through 12/31/2018.

After matching, we identified 21,942 patients who died from suicide (cases) and 21,942 controls. Cases were younger (by 1.4 years), more likely to be male, single or divorced, and non-Hispanic White. Additionally, cases were less likely to have a religious affiliation and more likely to have unknown LGBT status (Table 2).

**TABLE 2:** Patient characteristics between the case and control groups (United States, 2023).

	Case *n* = 21,942	Control *n* = 21,942	*p*-value
Age: mean (Std)	60.9 (16.2)	62.3 (18.3)	*p* < 0.001
Gender: n (%)			*p* < 0.001
Male	21,194 (97)	20,303 (93)	
Female	748 (3)	1,639 (7)	
Marital Status: n (%)			*p* < 0.001
Single	3,743 (17)	2,683 (12)	
Divorced	6,384 (29)	4,831 (22)	
Married	9,198 (42)	12,136 (55)	
Separated	742 (3)	674 (3)	
Widowed	1,545 (7)	1,455 (7)	
Unknown	330 (2)	163 (1)	
Race-Ethnicity: n (%)			
Non-Hispanic White	16,687 (76)	15,149 (69)	*p* < 0.001
Hispanic	796 (4)	1,314 (6)	
Non-Hispanic Black	1,007 (5)	3,560 (16)	
Other	2,021 (9)	1,578 (7)	
Unknown	1,431 (7)	341 (2)	
Religion: n (%)			*p* < 0.001
Baptist	2,764 (13)	3,906 (18)	
Other-Protestant	6,292 (29)	6,172 (28)	
Christian (Non-Specific)	1,199 (5)	1,210 (6)	
Catholic	3,678 (17)	4,505 (21)	
Other-Religion	1,414 (6)	1,352 (6)	
None/Unknown	6,595 (30)	4,797 (22)	
LGBT Status: n (%)			*p* = 0.057
Unknown	20,166 (92)	20,028 (91)	
Yes	575 (3)	628 (3)	
No	1,201 (5)	1,286 (6)	

**FIGURE 2:**
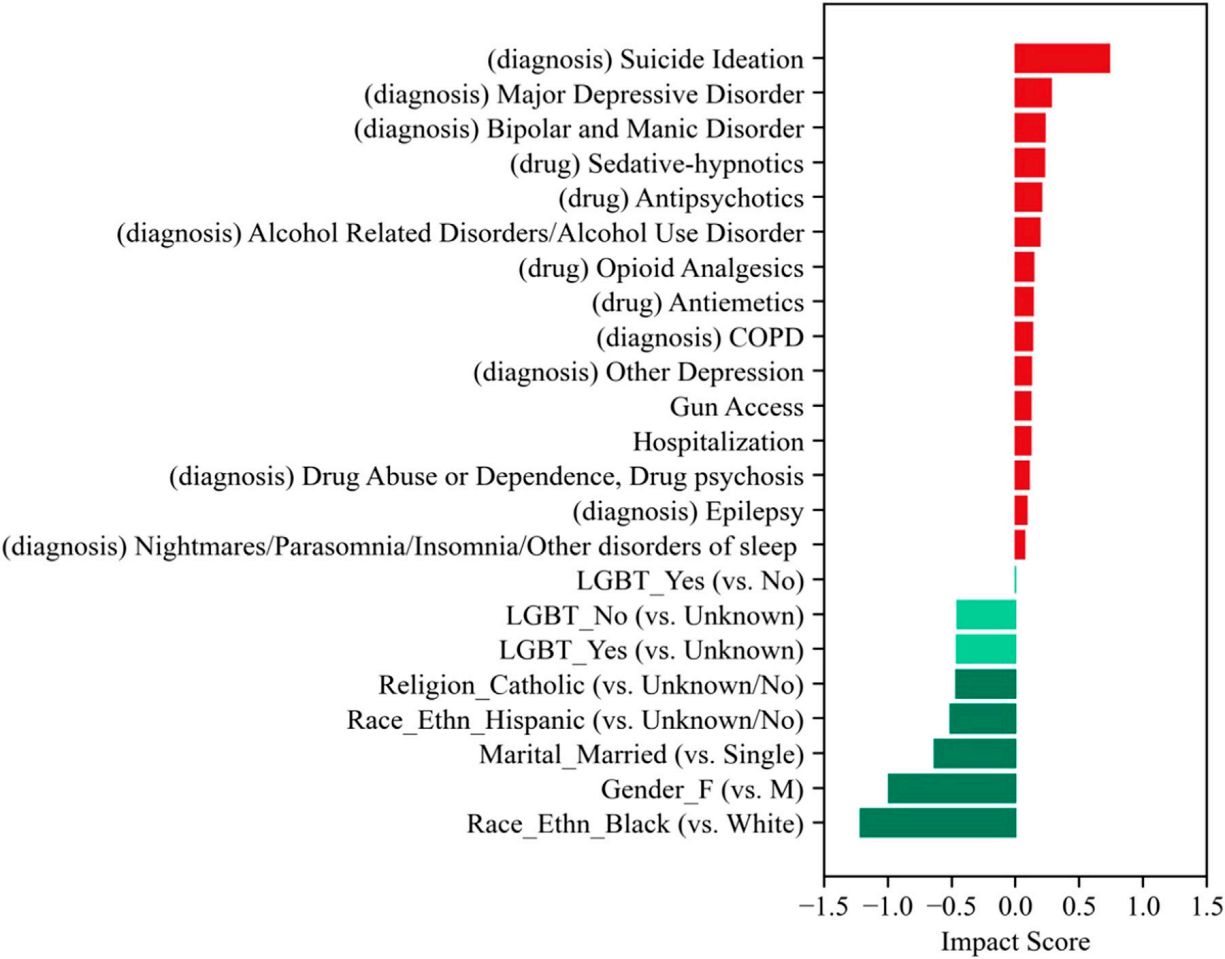
Features with top impact scores on the deep neural network model (United States, 2023).

The DNN model achieved an AUC of 76.7% and sensitivity and specificity both of 70.0% in classifying suicide death. We obtained similar AUC for training (77.6%) and validation (76.3%), showing no evidence of outfitting. Using our explainable AI, we evaluated the impact of each feature on the predicted outcome. Known risk factors for suicide death, such as suicide ideation, mental illness, and drug abuse, were among the top features in our model (Figure 2).

Our primary feature of interest (disclosed LGBT status) was found to be protective. Specifically, both disclosed LGBT and non-LGBT status was associated with lower risk than unknown LGBT status (impact score = −0.462 and −0.459, respectively). Patients with disclosed LGBT status had slightly lower risk than those who with disclosed non-LGBT status (impact score difference = −0.003) based on our analysis.

**FIGURE 3:**
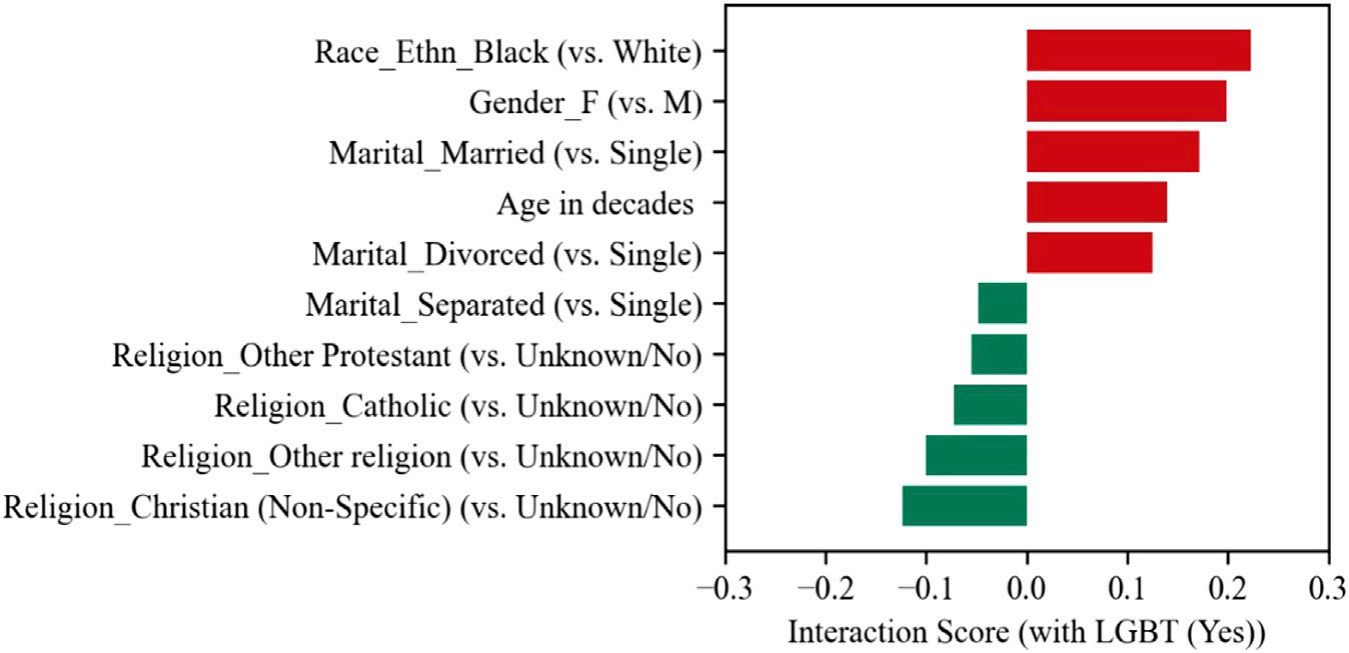
Features with top interaction scores with LGBT (Yes) Status (United States, 2023).

Additionally, disclosed LGBT status interacted with other features in the context of suicide mortality risk. For example, black, female, married, or older LGBT veterans appear to be at greater risk for suicide death. On the other hand, LGBT veterans of various religions and married but separated appear to be at lower risk (Figure 3).

## Discussion

As stated in the introduction, suicide claims tens of thousands of lives in the United States. Individuals identified as LGBT have a particularly high risk [2–5]. This, however, should not be interpreted as that sexual and gender minorities are inherently more prone to suicide. Minority stress associated with discrimination and bias, for example, is a key factor.

Predicting suicide death is extremely challenging due to its rarity. Machine learning (ML) is well-suited for this task because it can handle a large number of predictors and capture non-linear associations and interactions between variables. While using ML has enhanced prediction accuracy, these models still exhibit very low positive predictive values (PPVs) [37]. Additionally, a known limitation of ML models is their low clinical interpretability [38]. Our DNN model, which uses a case-control study design, has achieved a PPV of 69%. We are also able to provide additional insight into the model using explainable AI.

Like most observational studies, this study does not intend to draw causal conclusions [39]. Randomized controlled trials, which are the gold standard for providing causal inference, are not suitable for the present analysis, as the contemporary scientific evidence suggests that LGBT status is not modifiable. This analysis provides informative insights into the association between disclosed LGBT status and the risk of suicide death beyond structured EHR data, with adjustments for a comprehensive list of clinical factors. Moreover, the explainable AI interpretation is consistent with previous research findings, reassuring our confidence in the model’s accuracy and interpretability. Among 203 input features, many with highest or lowest model impacts have evidence in the literature. For example, suicide ideation often precede suicide attempt and death [40]. Mental illnesses such as depression and drug abuse are recognized as major risk factors for suicidality [41]. The link between gun access and suicide has been repeatedly reported [42]. Alternatively, certain religious affiliations and marital status have been found to be protective [43, 44].

After adjusting for other risk factors, we found that known LGBT status (positive and negative) was associated with reduced suicide risk compared to unknown LGBT status. Patients who were self-reported as LGBT had slightly lower risk of suicide death than those who were self-reported as non-LGBT, and both groups had lower risk than the unknown LGBT group. In other words, we found that documentation of SO/GI status does not confer an increase in suicide death when compared to patients of lacking SO/GI documentation, independent of other factors. This finding may seem surprising, but it is plausible with a closer look.

Lynch et al, reported “The crude suicide rate among sexual minority veterans (82.5 per 100,000 person-years) was higher than the rate in the general veteran population (37.7 per 100,000 person-years).” In our analysis, the all-cause mortality rate is 13% and the suicide mortality rate is 0.3% for LGBT group, which is similar to those reported by Lynch et al. (13% and 0.4%). The suicide mortality rate per 100,000 person-years is, however, much lower at 39.1. This is partly because we used the enrollment date as the index date and Lynch used the first documentation date as the index date. If we use our first documentation date of LGBT within our observation period (2009/10 to 2018/12), the rate is 76.6 which is much similar to Lynch’s number (82.5). Another reason is that our original dataset included more LGBT Veterans (231,032 vs. 96,896), due to the differences in the respective NLP methods. Both NLP tools [30, 45] achieved a PPV of 86%, while our tool had higher sensitivity (88% vs. 80%), which led to a higher number of LGBT individuals. In preparation of this manuscript, we double checked the NLP PPV: we randomly sampled another 300 LGBT individuals from our dataset, performed human review, and found the PPV to be 86%.

Our analysis also adjusted for a wide range of covariates. Our results confirmed our hypothesis that disclosed LGBT status alone is distally associated with suicide death, and other risk factors have more direct impacts on the outcome. There is no direct contradiction with the previous reports. First, we did not simply report the prevalence of suicide death. Rather, we adjusted for important covariates such as suicide ideation, depression, drug abuse, marital status, and religion. Second, we did not assume that people without disclosed LGBT status are not LGBT. Third, LGBT documentation is increasing over time [46] and the social pressure associated with LGBT is decreasing. Consequently, this study can be viewed as a second-generation disparities study [47], in that it identifies several risk factors that account for group differences in prevalence of the outcome. These modifiable risk factors are a key point around which interventions can be developed and tested to reduce the elevated prevalence of suicidal risk among LGBT individuals.

Additionally, it is known that patient engagement is critical to suicide prevention. Known LGBT status suggests a higher level of engagement than unknown status, which may suggest an informed presence bias [48, 49], i.e., more engagement in the health systems increases the opportunities for SO/GI documentation and more engagement likely means greater service utilization and more opportunities for care and suicide prevention services. The latter is a particularly important point within the context of VA, which has constructed a large infrastructure for suicide prevention (e.g., creating a national network of Suicide Prevention Coordinators, predictive analytics, and patient media outreach) [50].

In looking at the interaction scores, one may have the misconception that “LGBT marriage” is a risk factor. Please note that a positive interaction score indicates that the risk is more *or* the benefit is less than the additive impacts. In this case, disclosed LGBT status and being married are both beneficial factors. The positive interaction score means that the combined benefit is less than the sum of the individual benefits. A similar example is diet and exercise interventions on reducing ageing-related cognitive decline that, while individually shown to have positive effects [51, 52], lack strong evidence of additive effects [53] i.e., there may be a ceiling effect of the benefits.

### Conclusion

Our study suggests that disclosed LGBT status is not inherently associated with the risk for suicide; however, other factors (e.g., social pressures, depression and anxiety caused by stigma) can increase suicidal thinking and behaviors. We note that our findings could be an artifact of documentation. SO/GI data are more likely to be documented in mental health visits than primary care [36]. When SO/GI is documented, patients may be already in mental health treatment, which then reduces their risk of suicide. At the same time, we did adjust for mental health diagnoses and medications, and we also compared the disclosed LGBT status with the disclosed non-LGBT status. In general, more patient engagement, including inquiring about patients’ sexual orientation, can potentially help mitigate suicide risk.

### Limitations

We fitted a classification model, not a prediction model. The model fitting is moderately good (AUC = 76.7%), suggesting that the model does not explain all variations in the outcome. Suicide is a complex phenomenon; Our list of features is not comprehensive; other explanatory features for suicide death such as region or urban/rural status were not included in the initial feature selection. Military sexual trauma screener results are not included as the results are missing in most patients. Given the complexity of the outcome, despite using a relatively large number of features to parse the variance, omitted variable bias is a possibility because of limitations of data included in the EHR.

Our cohort is predominantly male and skewed toward older patients compared with the general U.S. population. Relying on ICD codes for suicidal ideation and attempt is highly inconsistent and likely underestimates the prevalence [54]. Lastly, misclassification bias of LGBT status is a threat with all methods attempting to glean a personal identity from second-hand evidence (i.e., clinical notes). In our cohort, approximately 91% of patients have an unknown LGBT status. This means that we do not have enough evidence to characterize those patients based on the EHR data. Since noting the SO/GI is not the required, the missingness is common. Additionally, patients with positive LGBT status may be more reluctant to disclosure their SO/GI to their healthcare practitioners [55]. Even if patients do disclose, practitioners may choose not to chart it if they believed it is irrelevant to the care at-hand. Still, this project’s strength was in preserving this group rather than making assumptions about the unknown group or omitting them wholly from the analysis.

### Future Work

In the report, the impacts of the temporal features are measured as a whole (presence vs. absence). We plan to investigate the time effect of those features. Also, we plan to carry our further analyses of the combinations of risk factors that are particularly dangerous. Moreover, we would like to conduct subgroup analyses in L, G, B, T, and other sexual minorities.
